# Disease outbreak in wildlife changes online sales of management items

**DOI:** 10.1016/j.onehlt.2025.100988

**Published:** 2025-02-04

**Authors:** Tomohiko Endo, Shinya Uryu, Keita Fukasawa, Jiefeng Kang, Takahiro Kubo

**Affiliations:** a Biodiversity Division, National Institute for Environmental Studies, 16-2 Onogawa, Tsukuba, Ibaraki 305-8506, Japan; b Tokushima University, University of Tokushima, 1-1 Minamijosanjima-cho, Tokushima 770-8502, Japan; c Graduate School of Global Environmental Studies, Sophia University, Sophia University, 7-1 Kioi-cho, Chiyoda-ku, Tokyo 102-8554, Japan

**Keywords:** Human wildlife conflict, Wildlife infections disease, Consumer behaviour, Online sales, Active management, Passive management

## Abstract

Infectious diseases of wildlife cause human health hazards and economic losses. However, it is unclear how outbreaks affect human behaviour in relation to countermeasures against human–wildlife conflict. To explore the effects of infectious disease outbreaks among wild boars on countermeasure choices, we analysed online auction data before and after an outbreak of classical swine fever in wild boar. Online sales of boar traps decreased by 17 % after the outbreak, whereas sales of control items increased by 73 %. These results imply that infectious disease outbreaks in wildlife shift people's countermeasures from active to passive management. Since active trapping for the control of wildlife populations is essential to the avoidance of human–wildlife conflict, our findings show that outbreaks of infectious disease can aggravate conflict. Governments, farmers and hunters need to improve population control after outbreaks of infectious disease.

## Highlights

• Few studies have explored infectious disease's impacts on consumer behaviour.
• We found wildlife disease outbreaks changed sales of countermeasures.
• Changing countermeasures from active to passive can increase human-wildlife conflicts.
• Understanding human behaviour change is required to enhance wildlife infection control.

## 1 Introduction

Infectious diseases of wildlife have caused serious challenges in conservation, wildlife management, public health and social economy [1]. More than 60 % of infectious diseases emerging in human populations for the first time are of zoonotic origin [2,3]. The infections shared between wildlife and livestock causes substantial economic losses and incurs substantial costs for biosecurity [4,5]. Furthermore, transmission of infectious diseases among multiple species not only threatens endangered species but also limits the productivity and density of wildlife, decreasing economic and recreational values [6,7].

Disease control depends heavily on peoples' behaviour and motivations, but different people have different values and backgrounds [8,9]. For instance, hunters ascribe commercial and recreational value to wildlife [7], whereas livestock farmers and veterinarians recognize wildlife as reservoirs of infectious diseases with the potential to cause economic losses and risks to public health [7,8]. Therefore, it is important to provide sufficient scientific evidence to avoid conflicts among stakeholders before implementing disease control and policy interventions [7].

To conduct effective wildlife management, including disease control, it is essential to combine several measures, such as reducing reservoir animal populations and preventing disease introduction [10,11]. However, a disease outbreak in wildlife can cause an imbalance in responses—in part because of the heterogeneity of peoples' values and backgrounds—that can interfere with wildlife management [8,9]. For example, if there is a risk of infection in humans, hunters have less motivation to hunt because they may want to mitigate the risk to themselves of infection [12,13]. But even if there is no risk, there is less motivation to trap wildlife owing to the decrease in populations due to disease and the extra need for disinfection following trapping [14,15]. Such reductions in hunting and trapping not only increase disease prevalence and duration in wildlife populations [16]: they also make fundamental population management more difficult [12]. Livestock farmers consider control measures such as fences important to biosecurity [17], but these are costly and must be maintained [11,18]. Moreover, when there is no evidence that measures are effective, or when costs are high, they lapse [19].

In Japan, wild boars (*Sus scrofa*) have spread nationwide over the past few decades, resulting in increased conflicts with people, such as crop damage and attacks on humans [20]. Following updates to the law in 2014 and the introduction of policies to promote culling, the estimated population of wild boars has continued to decline year by year [21]. A hunting licence is required to hunt or trap wild boars. However, the number of gun hunters has been decreasing in recent years, and about 60 % of wild boars are now caught in snare traps or box traps [22]. In addition, solid fences, electric fences and repellents that use sound, light and smell have been installed to control crop damage, and scientific verification of their effectiveness has been reported [23,24]. Against this backdrop, the first classical swine fever (CSF) outbreak in 26 years occurred in Gifu Prefecture in September 2018, and wild boars infected with CSF continue to be reported across Japan [25]. To counter the disease, culling measures involving guns and traps, control measures using solid fences and electric fences, and distribution of oral vaccines have been implemented. When the CSF outbreak first occurred, private gun hunting and trapping was temporarily banned in Gifu prefecture and nearby areas [26]. However, these regulations were lifted in 2020 as culling measures were promoted to control diseases [27]. Furthermore, these regulations were not applied in the other CSF infected areas, and culling measures were enhanced to decrease the wild boar population [28].

Previous epidemiological and ecological studies of disease control in wild boars included evaluation of the risk of transmission to domestic pigs and the efficiency of distribution of oral vaccines [29,30]. Other studies focused on changes in people's attitudes and perceptions about disease control [13,19], but not on changes in measures used to control wildlife disease. Lack of clarity on the effects of the CSF outbreak on the behaviour of people involved in wild boar control, such as hunters and farmers, might misguide policy design for effective disease control. Therefore, it is essential to develop effective disease control and wildlife management strategies through a transdisciplinary “One Health” approach that integrates existing knowledge with a social science perspective [31,32].

Here, we analysed changes in people's responses to CSF in wild boars in Japan to understand responses to an infectious disease that poses no risk of infection in humans. Our aim was to determine how the occurrence of CSF in wild boars affected people's control behaviour. Our findings highlight a challenge for wildlife management due to behaviour changes after the CSF outbreak.

## 2 Methods

### 2.1 Data collection

We focused on purchases of wild boar countermeasures to understand changes in people's preferences for wild boar control. Here, we define countermeasures as capture and prevention to mitigate conflicts between human and wildlife (i.e., boar in this study). Determination of consumer preferences through such purchasing behaviour has been used to examine social attitudes and intervention effects, especially in wildlife trade and wild meat use [33,34].

We analysed 9 years of purchase data, from January 2014 to December 2022, obtained from Yahoo Auctions, one of the largest online marketplaces in Japan. The data were downloaded from Aucfan, a mirror site that archives the history of the auction listings and purchases. We targeted the ‘Flower/Gardening’, ‘Housing/Interior’ and ‘Sports/Leisure’ categories, in which wildlife control items are sold on Yahoo Auctions. To trim the data, first we extracted all products with the word ‘boar’ (in Japanese) in the title. Then we excluded products that were not related to countermeasures (e.g., stickers, figurines). For each entry, the extracted data contained the sales category, title, seller ID, sales price, sales date and product description. We manually categorized the countermeasures into two categories according to previous studies [22,24]: trapping items (snare traps and box traps) and control items (solid fences, electric fences and repellents). Box traps and solid fences last 10 to 15 years, and snare traps and electric fences last 3 to 5 years [35,36]. Most of the repellents were single use.

### 2.2 Statistical analysis

We conducted a causal impact analysis by using a Bayesian Structural Time Series Model [37] to evaluate the causal effects of the CSF outbreak on the purchase of wild boar control items. The model predicts the numbers of countermeasure items purchased over time in a counterfactual scenario without CSF and compares them against the observed values, allowing the difference to be evaluated as an intervention effect. We defined January 2014 to August 2018 as the pre-intervention period (before the CSF outbreak) and September 2018 to December 2022 as the post-intervention period. We conducted two analyses to estimate how the occurrence of CSF in wild boars affected changes in the purchase of trapping items (snare traps, box traps; Model 1) and control items (fences, repellents; Model 2). In both models, the objective variable was the number of items per month, and the season (spring, Apr–Jun; summer, Jul–Sep; autumn, Oct–Dec; winter, Jan–Mar) was treated as a confounding effect. The significance of the CSF outbreaks was determined from posterior probability of causal effects. We conducted all analyses in R v. 4.2.2 software [38] and used the R package Causal Impact [37] for causal impact analysis.

## 3 Results

### 3.1 Purchases of wild boar countermeasure items

**Fig. 1:**
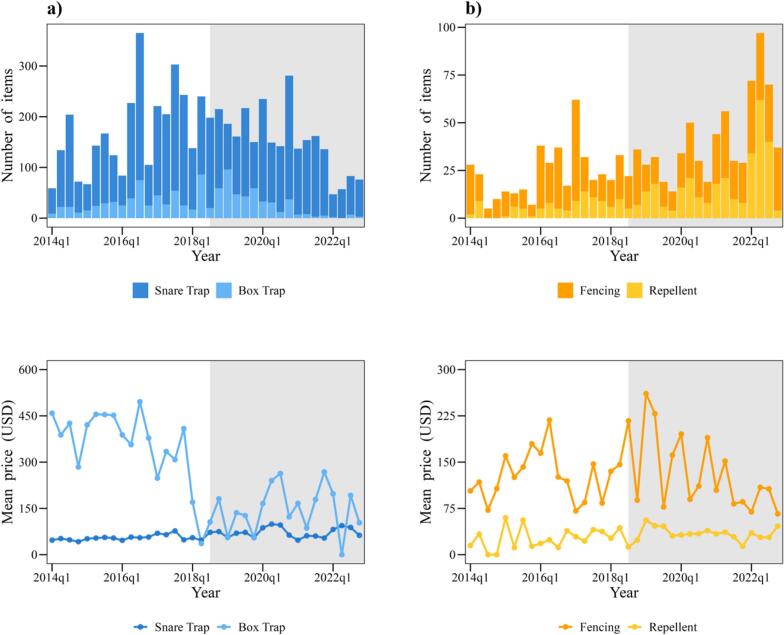
(Top) Numbers of items and (bottom) average price for each item. (Left) trapping items and (right) control items. Shading indicates period after CSF arrived.

From 2014 to 2022, 7032 wild boar control items were traded, mainly 4833 snare traps (68.73 %) and 1054 box traps (14.99 %), followed by 733 fences (10.42 %) and 412 repellents (5.86 %). Box traps were costliest (Mean trading price: 240 USD, calculated at 100 JPY = 1 USD), followed by fences (Mean trading price: 122 USD; Fig. 1).

### 3.2 Effects of CSF on wild boar countermeasure items

**Fig. 2:**
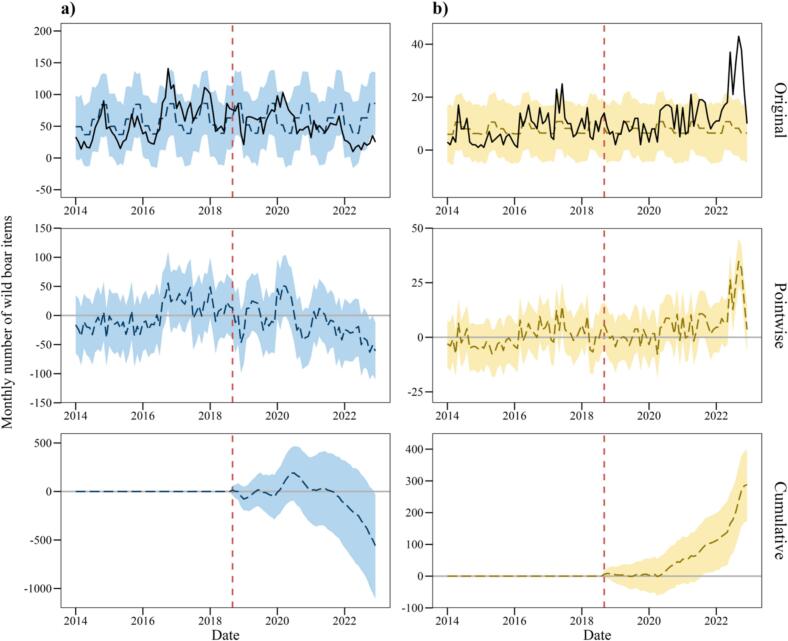
Effects of CSF on (a) trapping items and (b) control items as wild boar countermeasures. The vertical dashed red line shows the start of the CSF outbreak. “Original” shows the actual number (—) vs. the predicted number of purchases (− − -) under no outbreak. “Pointwise” shows the differences between actual and predicted monthly purchases. “Cumulative” shows the cumulative difference between actual and predicted monthly purchases. The coloured shading indicates the 95 % confidence interval. (For interpretation of the references to colour in this figure legend, the reader is referred to the web version of this article.)

The average number of monthly purchases of trapping items was 51 observed after the CSF outbreak but 62 predicted in the counterfactual scenario [95 % CI: 51–72] (Table 1). The cumulative value of the difference between monthly observed and predicted values was −17 % [95 % CI: −29 % to −0.67 %], indicating a negative causal effect of CSF that led to a relative decrease in the number of trapping items purchased (Fig. 2; Table 1).

**Table 1:** Results of Bayesian posterior inference in causal analysis of the impact of classical swine fever on wild boar countermeasure items.

	Model 1		Model 2	
	Number of trapping items (monthly)		Number of control items (monthly)	
	Average	Cumulative	Average	Cumulative
Actual	51	2647	13	697
Prediction (s.d.)	62 (5.2)	3205 (269.3)	7.9 (1.1)	408.8 (54.6)
95 % CI	[51, 72]	[2665, 3720]	[5.8, 9.9]	[304.2, 516.4]
Absolute effect (s.d.)	−11 (5.2)	−558 (269.3)	5.5 (1.1)	288.2 (54.6)
95 % CI	[−21, −0.34]	[−1073, −17.90]	[3.5, 7.6]	[180.6, 392.8]
Relative effect (s.d.)	−17 % (7.1 %)	−17 % (7.1 %)	73 % (24 %)	73 % (24 %)
95 % CI	[−29 %, −0.67 %]	[−29 %, −0.67 %]	[35 %, 129 %]	[35 %, 129 %]
Posterior tail-area probability *P*	0.022		0.001	
Posterior prob. of a causal effect	97.76 %		99.90 %	

In Model 1, the response variable was the monthly number of trapping items, and the quarterly seasonal effect was used as a covariate. In Model 2, the response variable was the monthly number of control items, and the quarterly seasonal effect was used as a covariate.

The average number of monthly purchases of control items was 13 observed after the CSF outbreak but 7.9 predicted [95 % CI: 5.8–9.9] (Table 1). The cumulative value of the difference between monthly observed and predicted values was 73 % [95 % CI: 35 %–129 %], indicating a positive causal effect of CSF that led to a relative increase in the number of control items purchased (Fig. 2; Table 1).

## 4 Discussion

It is important to understand the perceptions and behaviours of people involved in countermeasures when planning wildlife disease control [11,39]. We analysed changes in consumer preferences for countermeasure items from long-term market purchase data to clarify the effect of the CSF outbreak on people's behaviour. The findings suggest that the occurrence of CSF in wild boars reduced people's preference for trapping items and increased it for control items.

Culling has two roles in wildlife disease control. The first is to reduce the prevalence of host animal disease in combination with other disease control measures (e.g., vaccination) [11]. The second is to determine the infection status of the population from examination of the culled animals [11]. In wild boar CSF control, culling is used in addition to other disease control measures, such as oral vaccination [40]. In Japan, it is also conducted in combination with oral vaccination, and culled animals are used to evaluate the infection status and for surveillance and monitoring [28]. A hunting licence is required to cull wild boars, so the reduced preference for trapping measures in our results indicates a change in hunters' attitudes. The reduced preference for trapping may reduce the effectiveness of trapping in wild boar disease control. For example, it may decrease the number of infected animals culled and thus maintain a high prevalence in the population. In addition, there is a risk that the quality level of surveillance and monitoring to determine the status of infection will decline as the number of animals examined decreases. CSF does not pose a risk of transmission to humans. In Japan, based on information from the World Organization for Animal Health, the information that CSF does not infect humans is widely announced through the national and prefectural governments [25]. Therefore, it is unlikely that the reason for the decreased preference for trapping was to avoid the risk of infection among hunters [13]. On the other hand, the findings are consistent with reports that even if there is no risk of infection in humans, the reduced population density caused by the disease and the workload of disinfection during culling reduced the motivation to take countermeasures [14,15]. A further investigation of hunters' attitudes to and motivations for infectious disease outbreaks is required to understand how preferences change [41,42].

Our results indicate that the occurrence of CSF in wild boars increases the preference for control measures (Fig. 2; Table 1). Control measures are one of the most effective ways to reduce the cross-transmission of wildlife diseases between wildlife and livestock and have been used to strengthen biosecurity in barns to control diseases caused by wild boars [11,43]. The Japanese government mandated control measures (especially solid fences) for livestock farms in 2020 as countermeasures against CSF and other infectious diseases transmitted by wildlife [44]. This may have been one of the factors that promoted people's preferences for control measures. Control measures are widely used also to prevent wild boars from disturbing protected areas, pastures and agricultural lands, and have been reported to be highly effective when set up at an appropriate scale and at appropriate heights [45]. On the other hand, it is necessary to maintain the effectiveness of control by managing the surrounding vegetation and regularly checking for broken parts [46]. Therefore, disadvantages include the cost and effort involved in maintenance and management [45,47]. If preferences for culling decrease with infectious disease outbreaks and the wild boar population increases, sustainable control measures may become difficult because of long-term conflicts with wild boars and increased labour and costs of maintenance and management. Therefore, it is necessary to examine the cost-effectiveness and sustainability of the long-term implementation of control measures in situations where trapping measures are declining, through mathematical models and empirical studies [18].

Although we used unique online sales of wild boar countermeasure items, it is not possible to explore user heterogeneities in the online platform. Since the platform has, for example, private individuals and companies, comparing user composition with purchasing trends may give us a deeper understanding of the impact of wildlife diseases on human behaviour [48].

We also mention here the limitation that this study did not consider the impact of wildlife population dynamics. When a population decreases significantly owing to disease, for example, capture efforts can either increase as capturing becomes more difficult or decrease if capturing is abandoned [45,49]. Even if the population decreases significantly, human–wildlife conflicts do not disappear completely, so the purchase of control items can be maintained or increased. As causal inference approaches have developed rapidly even if wildlife management [50,51], further research is expected to apply such approaches to explore the relationship.

## 5 Conclusions

Population management is important where conflicts with people have occurred owing to animals' overabundance [6,52]. Our results suggest that infectious disease outbreaks in wildlife shift the countermeasure behaviour of people from active management, such as trapping, to passive management, creating an imbalance between culling and control measures. That is, the implementation of control measures alone does not directly affect the wildlife population. This poses the risk of a negative loop that escalates human–wildlife conflicts. Therefore, wildlife managers need to strengthen population management through policy interventions and behaviour change among hunters to maintain constant culling pressure. However, any such change in behaviour must be consistent with society's demand for non-lethal methods such as control measures [45,53]. Outbreaks of infectious diseases in wildlife may temporarily mitigate conflicts in people's intentions towards wildlife management. This may be desirable in the short term. However, it is difficult to rely only on non-lethal methods to manage wildlife in which conflicts with people have occurred [54]. Wildlife managers will need to design management plans for CSF control measures with a long-term perspective and for the future coexistence of wildlife and people. Our analysis of the purchasing behaviour of people involved in countermeasures showed that an outbreak of CSF may make it difficult not only to control the disease, but also indirectly to continue wildlife management. To solve this problem and achieve sustainable wildlife management, behavioural research on both wildlife and humans is essential. Furthermore, our results suggest the importance of a “One Health” approach, emphasizing the need for interdisciplinary collaboration among veterinarians, wildlife managers, and government officials to effectively address wildlife infectious diseases.

## Fundings

This resea was supported by JSPS KAKENHI (No. 23K20058).

## CRediT authorship contribution statement

**Tomohiko Endo:** Writing – review & editing, Writing – original draft, Visualization, Formal analysis, Conceptualization. **Shinya Uryu:** Writing – review & editing, Software, Methodology, Data curation. **Keita Fukasawa:** Writing – review & editing, Validation, Formal analysis, Data curation. **Jiefeng Kang:** Writing – review & editing, Visualization, Software, Data curation. **Takahiro Kubo:** Writing – review & editing, Supervision, Project administration, Methodology, Conceptualization.

## Declaration of competing interest

The authors have no declaration of competing interest to declare.

## Data Availability

Data will be made available on request.
